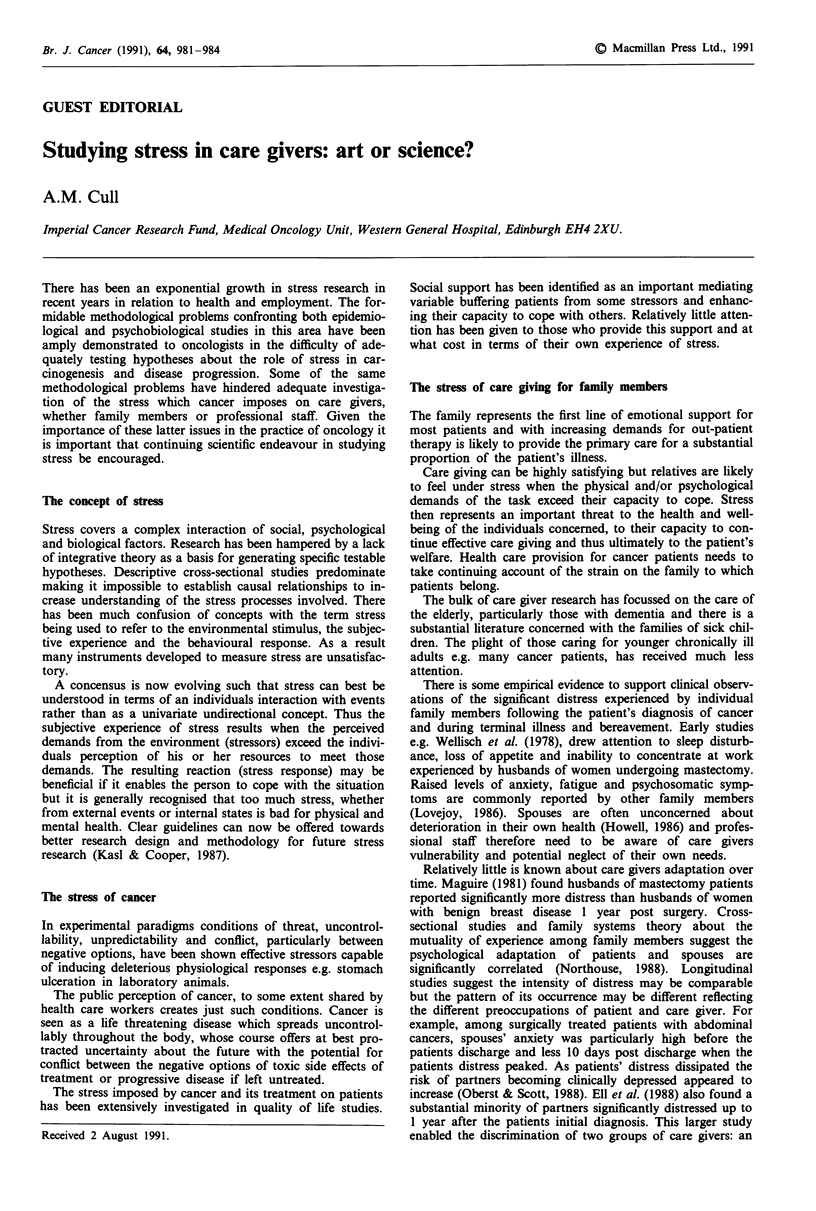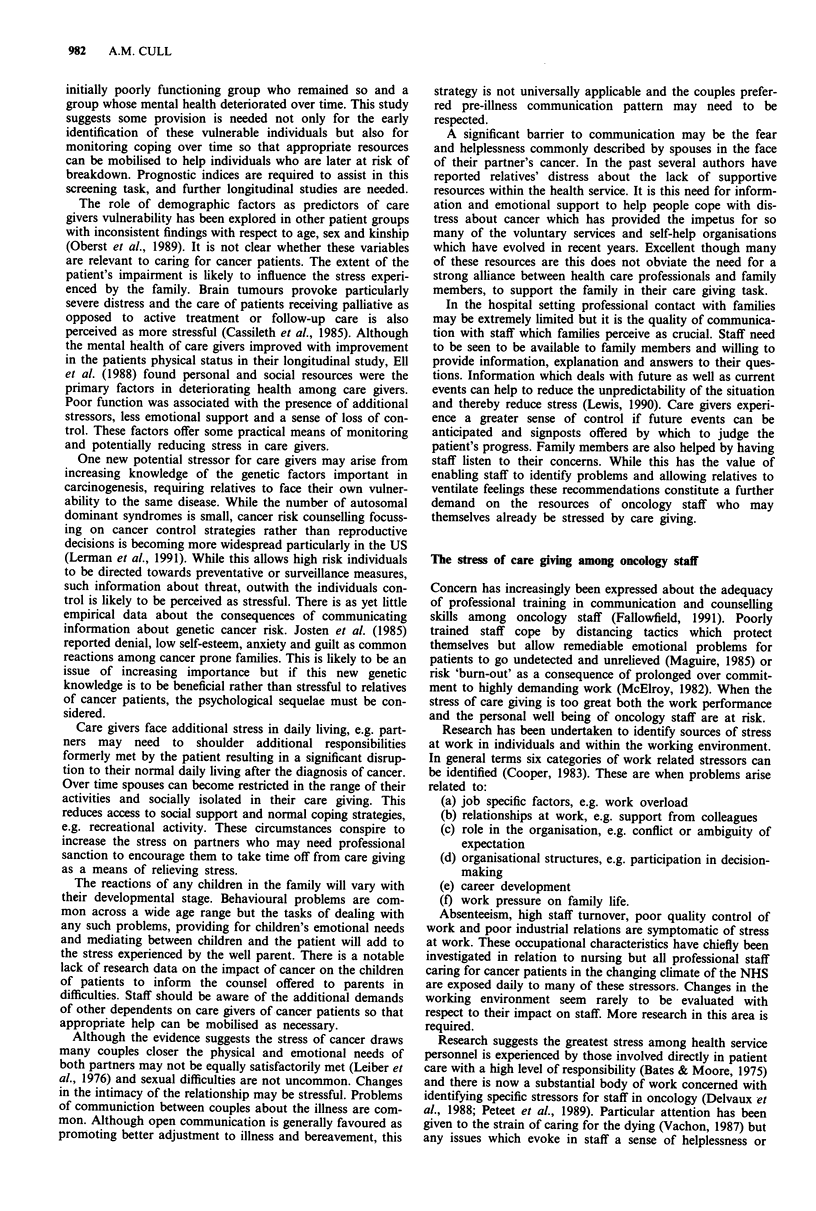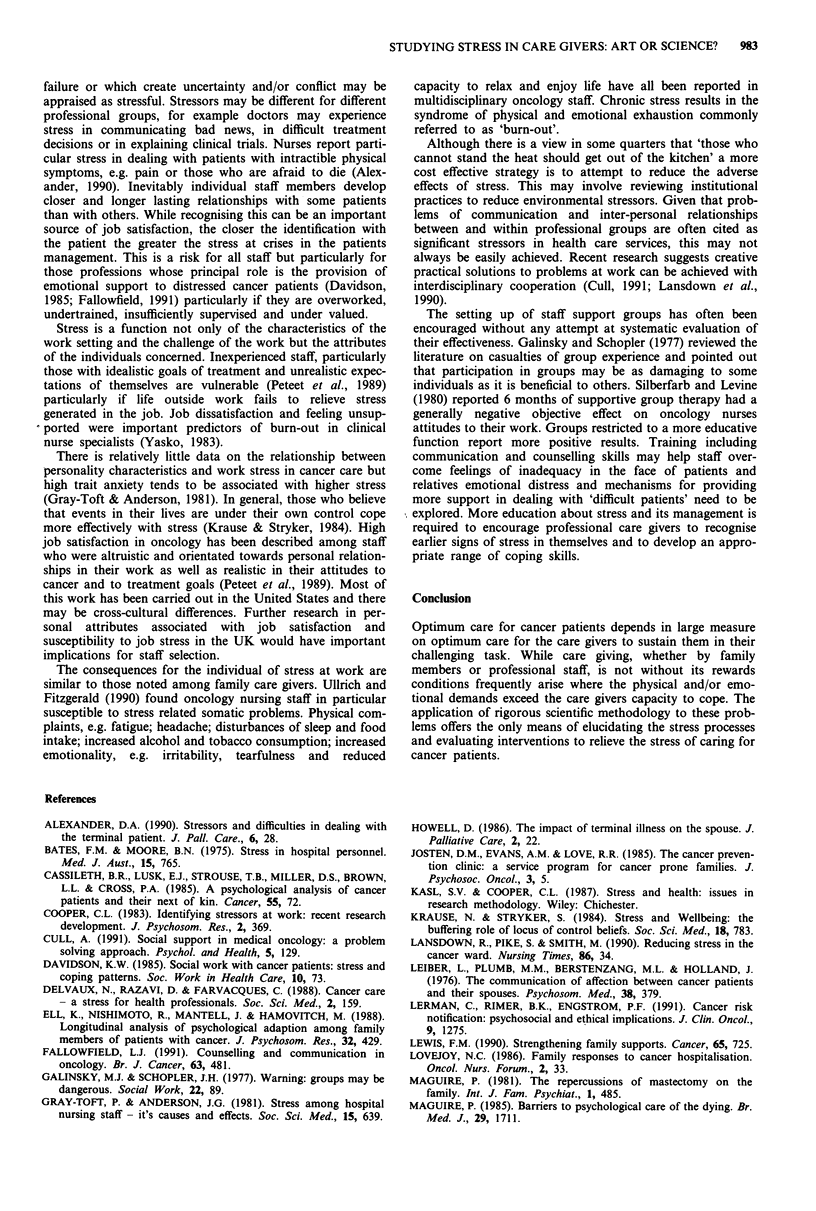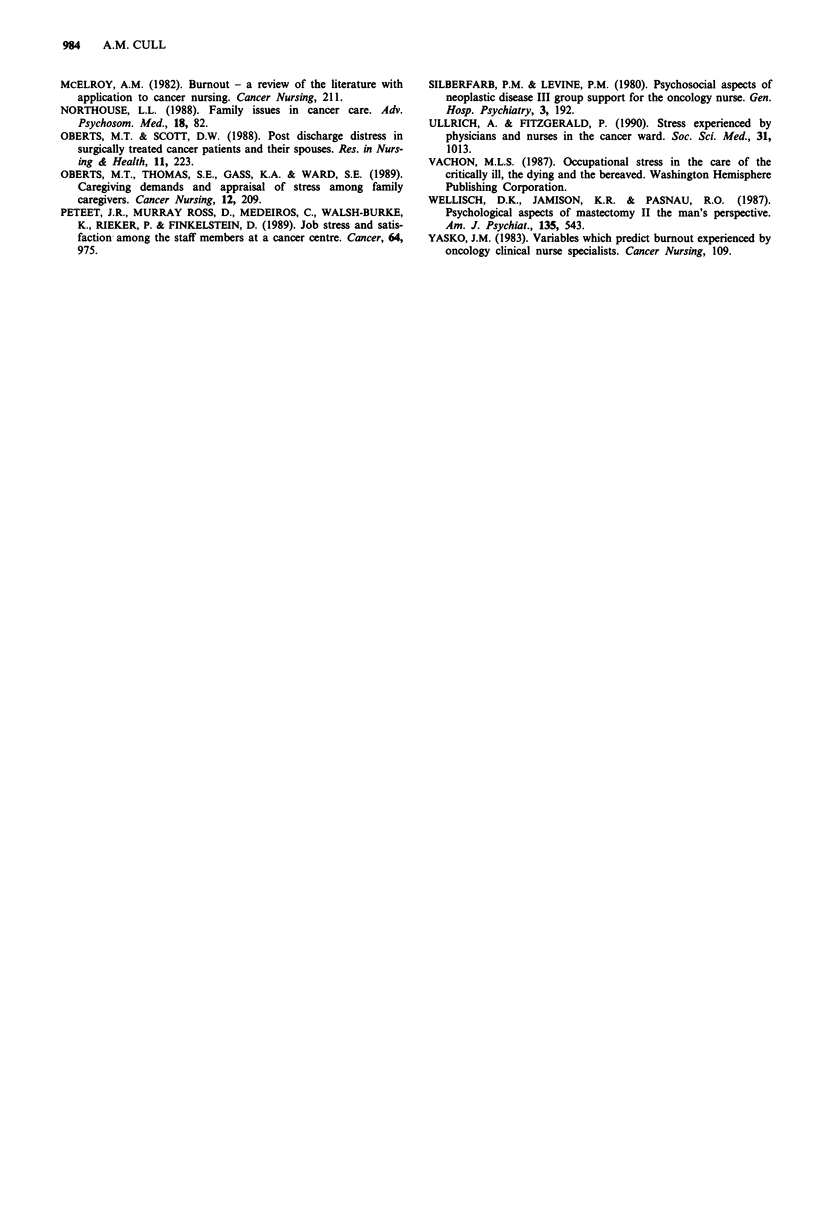# Studying stress in care givers: art or science?

**DOI:** 10.1038/bjc.1991.450

**Published:** 1991-12

**Authors:** A. M. Cull


*Br. J. Cancer* (1991), **64**, 981–984 © Macmillan Press Ltd., 1991

**GUEST EDITORIAL**

### Studying stress in care givers: art or science?

A.M. Cull

*Imperial Cancer Research Fund, Medical Oncology Unit, Western General Hospital, Edinburgh EH4 2XU.*

There has been an exponential growth in stress research in recent years in relation to health and employment. The formidable methodological problems confronting both epidemiological and psychobiological studies in this area have been amply demonstrated to oncologists in the difficulty of adequately testing hypotheses about the role of stress in carcinogenesis and disease progression. Some of the same methodological problems have hindered adequate investigation of the stress which cancer imposes on care givers, whether family members or professional staff. Given the importance of these latter issues in the practice of oncology it is important that continuing scientific endeavour in studying stress be encouraged.

### The concept of stress

Stress covers a complex interaction of social, psychological and biological factors. Research has been hampered by a lack of integrative theory as a basis for generating specific testable hypotheses. Descriptive cross-sectional studies predominate making it impossible to establish causal relationships to increase understanding of the stress processes involved. There has been much confusion of concepts with the term stress being used to refer to the environmental stimulus, the subjective experience and the behavioural response. As a result many instruments developed to measure stress are unsatisfactory.

A concensus is now evolving such that stress can best be understood in terms of an individuals interaction with events rather than as a univariate undirectional concept. Thus the subjective experience of stress results when the perceived demands from the environment (stressors) exceed the individuals perception of his or her resources to meet those demands. The resulting reaction (stress response) may be beneficial if it enables the person to cope with the situation but it is generally recognised that too much stress, whether from external events or internal states is bad for physical and mental health. Clear guidelines can now be offered towards better research design and methodology for future stress research (Kasl & Cooper, 1987).

### The stress of cancer

In experimental paradigms conditions of threat, uncontrollability, unpredictability and conflict, particularly between negative options, have been shown effective stressors capable of inducing deleterious physiological responses e.g. stomach ulceration in laboratory animals.

The public perception of cancer, to some extent shared by health care workers creates just such conditions. Cancer is seen as a life threatening disease which spreads uncontrollably throughout the body, whose course offers at best protracted uncertainty about the future with the potential for conflict between the negative options of toxic side effects of treatment or progressive disease if left untreated.

The stress imposed by cancer and its treatment on patients has been extensively investigated in quality of life studies. Social support has been identified as an important mediating variable buffering patients from some stressors and enhancing their capacity to cope with others. Relatively little attention has been given to those who provide this support and at what cost in terms of their own experience of stress.

### The stress of care giving for family members

The family represents the first line of emotional support for most patients and with increasing demands for out-patient therapy is likely to provide the primary care for a substantial proportion of the patient's illness.

Care giving can be highly satisfying but relatives are likely to feel under stress when the physical and/or psychological demands of the task exceed their capacity to cope. Stress then represents an important threat to the health and well-being of the individuals concerned, to their capacity to continue effective care giving and thus ultimately to the patient's welfare. Health care provision for cancer patients needs to take continuing account of the strain on the family to which patients belong.

The bulk of care giver research has focussed on the care of the elderly, particularly those with dementia and there is a substantial literature concerned with the families of sick children. The plight of those caring for younger chronically ill adults e.g. many cancer patients, has received much less attention.

There is some empirical evidence to support clinical observations of the significant distress experienced by individual family members following the patient's diagnosis of cancer and during terminal illness and bereavement. Early studies e.g. Wellisch *et al.* (1978), drew attention to sleep disturbance, loss of appetite and inability to concentrate at work experienced by husbands of women undergoing mastectomy. Raised levels of anxiety, fatigue and psychosomatic symptoms are commonly reported by other family members (Lovejoy, 1986). Spouses are often unconcerned about deterioration in their own health (Howell, 1986) and professional staff therefore need to be aware of care givers vulnerability and potential neglect of their own needs.

Relatively little is known about care givers adaptation over time. Maguire (1981) found husbands of mastectomy patients reported significantly more distress than husbands of women with benign breast disease 1 year post surgery. Cross-sectional studies and family systems theory about the mutuality of experience among family members suggest the psychological adaptation of patients and spouses are significantly correlated (Northouse, 1988). Longitudinal studies suggest the intensity of distress may be comparable but the pattern of its occurrence may be different reflecting the different preoccupations of patient and care giver. For example, among surgically treated patients with abdominal cancers, spouses' anxiety was particularly high before the patients discharge and less 10 days post discharge when the patients distress peaked. As patients' distress dissipated the risk of partners becoming clinically depressed appeared to increase (Oberst & Scott, 1988). Ell *et al.* (1988) also found a substantial minority of partners significantly distressed up to 1 year after the patients initial diagnosis. This larger study enabled the discrimination of two groups of care givers: an

Received 2 August 1991.

initially poorly functioning group who remained so and a group whose mental health deteriorated over time. This study suggests some provision is needed not only for the early identification of these vulnerable individuals but also for monitoring coping over time so that appropriate resources can be mobilised to help individuals who are later at risk of breakdown. Prognostic indices are required to assist in this screening task, and further longitudinal studies are needed.

The role of demographic factors as predictors of care givers vulnerability has been explored in other patient groups with inconsistent findings with respect to age, sex and kinship (Oberst *et al.*, 1989). It is not clear whether these variables are relevant to caring for cancer patients. The extent of the patient's impairment is likely to influence the stress experienced by the family. Brain tumours provoke particularly severe distress and the care of patients receiving palliative as opposed to active treatment or follow-up care is also perceived as more stressful (Cassileth *et al.*, 1985). Although the mental health of care givers improved with improvement in the patients physical status in their longitudinal study, Ell *et al.* (1988) found personal and social resources were the primary factors in deteriorating health among care givers. Poor function was associated with the presence of additional stressors, less emotional support and a sense of loss of control. These factors offer some practical means of monitoring and potentially reducing stress in care givers.

One new potential stressor for care givers may arise from increasing knowledge of the genetic factors important in carcinogenesis, requiring relatives to face their own vulnerability to the same disease. While the number of autosomal dominant syndromes is small, cancer risk counselling focussing on cancer control strategies rather than reproductive decisions is becoming more widespread particularly in the US (Lerman *et al.*, 1991). While this allows high risk individuals to be directed towards preventative or surveillance measures, such information about threat, outwith the individuals control is likely to be perceived as stressful. There is as yet little empirical data about the consequences of communicating information about genetic cancer risk. Josten *et al.* (1985) reported denial, low self-esteem, anxiety and guilt as common reactions among cancer prone families. This is likely to be an issue of increasing importance but if this new genetic knowledge is to be beneficial rather than stressful to relatives of cancer patients, the psychological sequelae must be considered.

Care givers face additional stress in daily living, e.g. partners may need to shoulder additional responsibilities formerly met by the patient resulting in a significant disruption to their normal daily living after the diagnosis of cancer. Over time spouses can become restricted in the range of their activities and socially isolated in their care giving. This reduces access to social support and normal coping strategies, e.g. recreational activity. These circumstances conspire to increase the stress on partners who may need professional sanction to encourage them to take time off from care giving as a means of relieving stress.

The reactions of any children in the family will vary with their developmental stage. Behavioural problems are common across a wide age range but the tasks of dealing with any such problems, providing for children's emotional needs and mediating between children and the patient will add to the stress experienced by the well parent. There is a notable lack of research data on the impact of cancer on the children of patients to inform the counsel offered to parents in difficulties. Staff should be aware of the additional demands of other dependents on care givers of cancer patients so that appropriate help can be mobilised as necessary.

Although the evidence suggests the stress of cancer draws many couples closer the physical and emotional needs of both partners may not be equally satisfactorily met (Leiber *et al.*, 1976) and sexual difficulties are not uncommon. Changes in the intimacy of the relationship may be stressful. Problems of communiction between couples about the illness are common. Although open communication is generally favoured as promoting better adjustment to illness and bereavement, this strategy is not universally applicable and the couples preferred pre-illness communication pattern may need to be respected.

A significant barrier to communication may be the fear and helplessness commonly described by spouses in the face of their partner's cancer. In the past several authors have reported relatives' distress about the lack of supportive resources within the health service. It is this need for information and emotional support to help people cope with distress about cancer which has provided the impetus for so many of the voluntary services and self-help organisations which have evolved in recent years. Excellent though many of these resources are this does not obviate the need for a strong alliance between health care professionals and family members, to support the family in their care giving task.

In the hospital setting professional contact with families may be extremely limited but it is the quality of communication with staff which families perceive as crucial. Staff need to be seen to be available to family members and willing to provide information, explanation and answers to their questions. Information which deals with future as well as current events can help to reduce the unpredictability of the situation and thereby reduce stress (Lewis, 1990). Care givers experience a greater sense of control if future events can be anticipated and signposts offered by which to judge the patient's progress. Family members are also helped by having staff listen to their concerns. While this has the value of enabling staff to identify problems and allowing relatives to ventilate feelings these recommendations constitute a further demand on the resources of oncology staff who may themselves already be stressed by care giving.

### The stress of care giving among oncology staff

Concern has increasingly been expressed about the adequacy of professional training in communication and counselling skills among oncology staff (Fallowfield, 1991). Poorly trained staff cope by distancing tactics which protect themselves but allow remediable emotional problems for patients to go undetected and unrelieved (Maguire, 1985) or risk 'burn-out' as a consequence of prolonged over commitment to highly demanding work (McElroy, 1982). When the stress of care giving is too great both the work performance and the personal well being of oncology staff are at risk.

Research has been undertaken to identify sources of stress at work in individuals and within the working environment. In general terms six categories of work related stressors can be identified (Cooper, 1983). These are when problems arise related to:

(a) job specific factors, e.g. work overload
(b) relationships at work, e.g. support from colleagues
(c) role in the organisation, e.g. conflict or ambiguity of expectation
(d) organisational structures, e.g. participation in decision-making
(e) career development
(f) work pressure on family life.

Absenteeism, high staff turnover, poor quality control of work and poor industrial relations are symptomatic of stress at work. These occupational characteristics have chiefly been investigated in relation to nursing but all professional staff caring for cancer patients in the changing climate of the NHS are exposed daily to many of these stressors. Changes in the working environment seem rarely to be evaluated with respect to their impact on staff. More research in this area is required.

Research suggests the greatest stress among health service personnel is experienced by those involved directly in patient care with a high level of responsibility (Bates & Moore, 1975) and there is now a substantial body of work concerned with identifying specific stressors for staff in oncology (Delvaux *et al.*, 1988; Peteet *et al.*, 1989). Particular attention has been given to the strain of caring for the dying (Vachon, 1987) but any issues which evoke in staff a sense of helplessness or failure or which create uncertainty and/or conflict may be appraised as stressful. Stressors may be different for different professional groups, for example doctors may experience stress in communicating bad news, in difficult treatment decisions or in explaining clinical trials. Nurses report particular stress in dealing with patients with intractible physical symptoms, e.g. pain or those who are afraid to die (Alexander, 1990). Inevitably individual staff members develop closer and longer lasting relationships with some patients than with others. While recognising this can be an important source of job satisfaction, the closer the identification with the patient the greater the stress at crises in the patients management. This is a risk for all staff but particularly for those professions whose principal role is the provision of emotional support to distressed cancer patients (Davidson, 1985; Fallowfield, 1991) particularly if they are overworked, undertrained, insufficiently supervised and under valued.

Stress is a function not only of the characteristics of the work setting and the challenge of the work but the attributes of the individuals concerned. Inexperienced staff, particularly those with idealistic goals of treatment and unrealistic expectations of themselves are vulnerable (Peteet *et al.*, 1989) particularly if life outside work fails to relieve stress generated in the job. Job dissatisfaction and feeling unsupported were important predictors of burn-out in clinical nurse specialists (Yasko, 1983).

There is relatively little data on the relationship between personality characteristics and work stress in cancer care but high trait anxiety tends to be associated with higher stress (Gray-Toft & Anderson, 1981). In general, those who believe that events in their lives are under their own control cope more effectively with stress (Krause & Stryker, 1984). High job satisfaction in oncology has been described among staff who were altruistic and orientated towards personal relationships in their work as well as realistic in their attitudes to cancer and to treatment goals (Peteet *et al.*, 1989). Most of this work has been carried out in the United States and there may be cross-cultural differences. Further research in personal attributes associated with job satisfaction and susceptibility to job stress in the UK would have important implications for staff selection.

The consequences for the individual of stress at work are similar to those noted among family care givers. Ullrich and Fitzgerald (1990) found oncology nursing staff in particular susceptible to stress related somatic problems. Physical complaints, e.g. fatigue; headache; disturbances of sleep and food intake; increased alcohol and tobacco consumption; increased emotionality, e.g. irritability, tearfulness and reduced capacity to relax and enjoy life have all been reported in multidisciplinary oncology staff. Chronic stress results in the syndrome of physical and emotional exhaustion commonly referred to as 'burn-out'.

Although there is a view in some quarters that 'those who cannot stand the heat should get out of the kitchen' a more cost effective strategy is to attempt to reduce the adverse effects of stress. This may involve reviewing institutional practices to reduce environmental stressors. Given that problems of communication and inter-personal relationships between and within professional groups are often cited as significant stressors in health care services, this may not always be easily achieved. Recent research suggests creative practical solutions to problems at work can be achieved with interdisciplinary cooperation (Cull, 1991; Lansdown *et al.*, 1990).

The setting up of staff support groups has often been encouraged without any attempt at systematic evaluation of their effectiveness. Galinsky and Schopler (1977) reviewed the literature on casualties of group experience and pointed out that participation in groups may be as damaging to some individuals as it is beneficial to others. Silberfarb and Levine (1980) reported 6 months of supportive group therapy had a generally negative objective effect on oncology nurses attitudes to their work. Groups restricted to a more educative function report more positive results. Training including communication and counselling skills may help staff overcome feelings of inadequacy in the face of patients and relatives emotional distress and mechanisms for providing more support in dealing with 'difficult patients' need to be explored. More education about stress and its management is required to encourage professional care givers to recognise earlier signs of stress in themselves and to develop an appropriate range of coping skills.

### Conclusion

Optimum care for cancer patients depends in large measure on optimum care for the care givers to sustain them in their challenging task. While care giving, whether by family members or professional staff, is not without its rewards conditions frequently arise where the physical and/or emotional demands exceed the care givers capacity to cope. The application of rigorous scientific methodology to these problems offers the only means of elucidating the stress processes and evaluating interventions to relieve the stress of caring for cancer patients.

### References

ALEXANDER, D.A. (1990). Stressors and difficulties in dealing with the terminal patient. *J. Pall. Care.*, **6**, 28.

BATES, F.M. & MOORE, B.N. (1975). Stress in hospital personnel. *Med. J. Aust.*, **15**, 765.

CASSILETH, B.R., LUSK, E.J., STROUSE, T.B., MILLER, D.S., BROWN, L.L. & CROSS, P.A. (1985). A psychological analysis of cancer patients and their next of kin. *Cancer*, **55**, 72.

COOPER, C.L. (1983). Identifying stressors at work: recent research development. *J. Psychosom. Res.*, **2**, 369.

CULL, A. (1991). Social support in medical oncology: a problem solving approach. *Psychol. and Health*, **5**, 129.

DAVIDSON, K.W. (1985). Social work with cancer patients: stress and coping patterns. *Soc. Work in Health Care*, **10**, 73.

DELVAUX, N., RAZAVI, D. & FARVACQUES, C. (1988). Cancer care – a stress for health professionals. *Soc. Sci. Med.*, **2**, 159.

ELL, K., NISHIMOTO, R., MANTELL, J. & HAMOVITCH, M. (1988). Longitudinal analysis of psychological adaption among family members of patients with cancer. *J. Psychosom. Res.*, **32**, 429.

FALLOWFIELD, L.J. (1991). Counselling and communication in oncology. *Br. J. Cancer*, **63**, 481.

GALINSKY, M.J. & SCHOPLER, J.H. (1977). Warning: groups may be dangerous. *Social Work*, **22**, 89.

GRAY-TOFT, P. & ANDERSON, J.G. (1981). Stress among hospital nursing staff – it's causes and effects. *Soc. Sci. Med.*, **15**, 639.

HOWELL, D. (1986). The impact of terminal illness on the spouse. *J. Palliative Care*, **2**, 22.

JOSTEN, D.M., EVANS, A.M. & LOVE, R.R. (1985). The cancer prevention clinic: a service program for cancer prone families. *J. Psychosoc. Oncol.*, **3**, 5.

KASL, S.V. & COOPER, C.L. (1987). Stress and health: issues in research methodology. Wiley: Chichester.

KRAUSE, N. & STRYKER, S. (1984). Stress and Wellbeing: the buffering role of locus of control beliefs. *Soc. Sci. Med.*, **18**, 783.

LANSDOWN, R., PIKE, S. & SMITH, M. (1990). Reducing stress in the cancer ward. *Nursing Times*, **86**, 34.

LEIBER, L., PLUMB, M.M., BERSTENZANG, M.L. & HOLLAND, J. (1976). The communication of affection between cancer patients and their spouses. *Psychosom. Med.*, **38**, 379.

LERMAN, C., RIMER, B.K., ENGSTROM, P.F. (1991). Cancer risk notification: psychosocial and ethical implications. *J. Clin. Oncol.*, **9**, 1275.

LEWIS, F.M. (1990). Strengthening family supports. *Cancer*, **65**, 725.

LOVEJOY, N.C. (1986). Family responses to cancer hospitalisation. *Oncol. Nurs. Forum.*, **2**, 33.

MAGUIRE, P. (1981). The repercussions of mastectomy on the family. *Int. J. Fam. Psychiat.*, **1**, 485.

MAGUIRE, P. (1985). Barriers to psychological care of the dying. *Br. Med. J.*, **29**, 1711.

McELROY, A.M. (1982). Burnout – a review of the literature with application to cancer nursing. *Cancer Nursing*, 211.

NORTHOUSE, L.L. (1988). Family issues in cancer care. *Adv. Psychosom. Med.*, **18**, 82.

OBERTS, M.T. & SCOTT, D.W. (1988). Post discharge distress in surgically treated cancer patients and their spouses. *Res. in Nursing & Health*, **11**, 223.

OBERTS, M.T., THOMAS, S.E., GASS, K.A. & WARD, S.E. (1989). Caregiving demands and appraisal of stress among family caregivers. *Cancer Nursing*, **12**, 209.

PETEET, J.R., MURRAY ROSS, D., MEDEIROS, C., WALSH-BURKE, K., RIEKER, P. & FINKELSTEIN, D. (1989). Job stress and satisfaction among the staff members at a cancer centre. *Cancer*, **64**, 975.

SILBERFARB, P.M. & LEVINE, P.M. (1980). Psychosocial aspects of neoplastic disease III group support for the oncology nurse. *Gen. Hosp. Psychiatry*, **3**, 192.

ULLRICH, A. & FITZGERALD, P. (1990). Stress experienced by physicians and nurses in the cancer ward. *Soc. Sci. Med.*, **31**, 1013.

VACHON, M.L.S. (1987). Occupational stress in the care of the critically ill, the dying and the bereaved. Washington Hemisphere Publishing Corporation.

WELLISCH, D.K., JAMISON, K.R. & PASNAU, R.O. (1987). Psychological aspects of mastectomy II the man's perspective. *Am. J. Psychiat.*, **135**, 543.

YASKO, J.M. (1983). Variables which predict burnout experienced by oncology clinical nurse specialists. *Cancer Nursing*, 109.